# Depression and Anxiety in Patients With Irreversible Vision Loss: Meta-Analysis and Systematic Review

**DOI:** 10.1177/00912174251382653

**Published:** 2025-10-08

**Authors:** Nirmit Shah, Edward Tran, Mohamed Aly, Vivian Phu, Ellie Laughlin, Monali S. Malvankar-Mehta

**Affiliations:** 1 Melbourne Medical School, 85084 The University of Melbourne, Melbourne, VIC, Australia; 2 Schulich School of Medicine and Dentistry, 70384 Western University, London, ON, Canada; 3 149991 Rocky Vista University College of Osteopathic Medicine, Parker, CO, USA; 4 Department of Epidemiology and Biostatistics, Schulich School of Medicine and Dentistry, 70384 Western University, London, ON, Canada; 5 Department of Ophthalmology, Schulich School of Medicine and Dentistry, 70384 Western University, London, ON, Canada

**Keywords:** irreversible vision loss, depression, anxiety, age-related macular degeneration (AMD), glaucoma, diabetic retinopathy, mental health, vision rehabilitation

## Abstract

**Objective:**

Approximately 295 million individuals globally live with moderate to severe irreversible vision loss, primarily due to conditions such as glaucoma, diabetic retinopathy, and age-related macular degeneration (AMD). Vision impairment diminishes quality of life leading to higher rates of depression and anxiety. This study investigated the prevalence of anxiety and depression in patients with irreversible vision loss, with a comparative analysis across the conditions of AMD, diabetic retinopathy, and glaucoma.

**Methods:**

A comprehensive literature search was conducted in Medline, Embase, CINAHL, and Cochrane databases, supplemented by manual searches of conference literature.

**Results:**

The prevalence of depression in patients with irreversible vision loss was found to be 21% (95% CI: 0.17-0.26) among 76 561 patients, with variations based on the cause: 27% (95% CI: 0.19-0.35) in AMD, 48% (95% CI: 0.32-0.64) in diabetic retinopathy, and 23% (95% CI: 0.16-0.29) in glaucoma. Anxiety prevalence was 22% (95% CI: 0.15-0.30) among 25 616 patients.

**Conclusion:**

The high prevalence of depression and anxiety underscores the need for comprehensive healthcare approaches that incorporate mental health support, including vision rehabilitation, psychotherapy, pharmacological interventions, and lifestyle modifications. Future research should explore factors that protect against anxiety and depression, as well as address the long-term effects of vision loss treatments on mental health outcomes.

## Introduction

A recent study addressing the Global Burden of Disease revealed that 295 million individuals currently have moderate to severe vision loss.^1^ The individuals who are most adversely impacted are those with irreversible vision loss affected by disease processes such as glaucoma, diabetic retinopathy, age-related macular degeneration (AMD), or uveal melanoma. Of these, AMD is found to be the most common cause of irreversible, moderate-to-severe vision loss, while glaucoma is the most common cause of irreversible blindness.^1^

The quality of life (QoL) for persons with visual impairments, as measured by the EQ-5D, has been demonstrated to be lower in this group than for many other chronic conditions including coronary syndrome, type-2 diabetes, and hearing impairments.^2^ Common instrumental activities of daily living, specifically reading and social interactions, are reported as substantially disturbed, contributing to an overall diminished QoL.^3^ These limitations placed on individuals with vision loss decrease their independence, making the requirement for long-term care significantly more likely.^4^

However, there are ways to overcome these limitations using vision rehabilitation programs. For example, researchers indicate that supportive training in adaptive oral hygiene programs and in using of low vision devices may be effective in improving day-to-day functioning and QoL.^5^ In addition, with the aid of low vision devices, patients found significant improvement in social functioning with others, also positively influencing QoL.^6^ Beyond the functional challenges of navigating daily life with vision impairments, there exists a component of emotional burden that is placed upon individuals. This emotional impact is notable in the incidence of depression observed in low-vision patients.^7^ We hypothesize that patients with irreversible vision loss are more likely to experience anxiety and depression when compared to individuals without significant visual impairments. Furthermore, the objective of this paper is to investigate the incidence of anxiety and depression in patients experiencing irreversible vision loss compared to non-vision loss individuals and evaluate the size of this effect.

## Methods

### Search Strategy

This systematic review adheres to the PRISMA (Preferred Reporting Items for Systematic Reviews and Meta-Analyses) guidelines. The comprehensive PRISMA checklist is available on request. On March 17, 2022, we conducted comprehensive searches in Medline (OVID), Embase (OVID), CINAHL, and Cochrane databases. Upon request, our search strategy, including the terminology used to identify articles about various causes of vision loss as well as different anxiety and mood disorders is also available. Gray literature searches involved submissions to conferences including the Association for Research in Vision and Ophthalmology (ARVO), American Academy of Ophthalmology (AAO), and Canadian Ophthalmological Society (COS) to find relevant poster presentations or abstracts.

### Inclusion Criteria

We investigated studies involving patients with irreversible causes of vision loss or blindness, including but not limited to glaucoma, age-related macular degeneration, and diabetic retinopathy, to assess the impact on mood disorders such as depression or anxiety. We included studies that provided objective scales for depression or anxiety, such as GAD-7 or PHQ-9. Conference abstracts were included if they provided adequate study details and data. We included clinical trials, cohort studies, comparative studies, economic analyses, multicenter studies, observational studies, and randomized controlled trials. Review articles, conference abstracts, case reports, systematic reviews, meta-analyses, letters to editors, and commentaries were excluded. Studies focusing on patients with reversible causes of vision loss, such as refractive errors, cataracts, corneal blindness, or early diabetic macular edema, were also excluded.

### Exclusion Criteria

We excluded articles that discussed patients with reversible causes of vision loss or blindness, including but not limited to refractive errors, cataracts, corneal blindness, or early diabetic macular edema. We excluded review articles, systematic reviews, meta-analyses, letters to editors, and commentaries. Single-patient case reports and studies involving additional interventions or treatments were also excluded. Conference abstracts were not considered if they did not provide sufficient study details and data. To avoid potential inconsistencies during translation, studies without an English copy were omitted. We placed no restrictions on publication year or geographical settings.

### Study Selection

**Figure 1.**
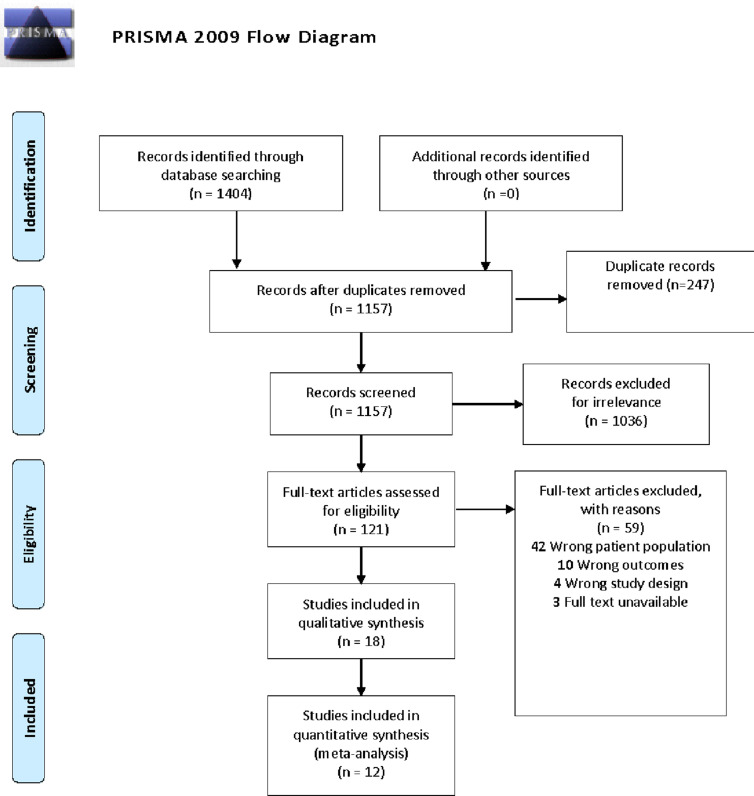
PRISMA Flow Chart Summarizing Search Results

The screening process was divided into three distinct levels: Level 1 - title, Level 2 - abstract, and Level 3 - full-text screening. Compiled literature was imported into Covidence, which automatically performs a duplication check to ensure each entry is unique. An additional manual review was conducted. The initial Level 1 and Level 2 screenings established inclusion and exclusion criteria. Level 3 screenings specifically evaluated mood disorders such as depression and anxiety in patients with irreversible causes of vision loss or blindness. To quantify the agreement between reviewers during the title, abstract, and full-text screening processes, Cohen’s kappa (κ) coefficient was calculated. Figure 1, titled “The PRISMA Flowchart,” visually represents the number of studies included and excluded at each level of the screening process.

### Risk of Bias

Risk of bias assessment was completed using the modified Downs and Black checklist.^8^ Studies scoring 20 or above, were deemed high quality, while those with scores ranging from 15 to 19 were categorized as medium quality, and studies scoring below 15 were labelled as poor quality. We excluded any studies scoring below 15.

### Statistical Analysis

Our meta-analysis was conducted using STATA 14.0 software (STATA Corporation, College Station, TX). We utilized the proportion as the measure for effect size or treatment effect. The I^2^ value was employed to gauge the heterogeneity among the studies, indicating the percentage of variation across studies attributable to heterogeneity rather than mere chance. The Chi-squared test helped determine if observed variances between studies were likely random occurrences. A significant Chi-squared statistic, coupled with a low *P*-value relative to its degree of freedom, signaled the existence of heterogeneity. Depending on the detected heterogeneity level, we employed either fixed-effect or random-effect models. To represent the findings and inspect potential publication bias, we generated forest and funnel plots.

## Results

### Search Results

Searches of online databases and gray literature yielded 1157 results after duplicates were removed. Following title and abstract screening, 1036 articles were deemed irrelevant, leaving 121 articles for full-text screening. Of these, 62 articles advanced to data extraction. All 62 studies were included in qualitative analysis. 13 studies were excluded from the quantitative analysis.^6,9-20^ due to limitations in the data provided. 59 studies were excluded from data extraction for not providing data consistent with other papers. Studies that were excluded did not meet the relevance criteria or failed to progress past the screening stages. The Cohen’s kappa (κ) coefficient for the title and abstract screening was 93%, and for the full-text screening, it was 21%.

### Study Characteristics

Table 1 details the demographic characteristics of the 62 studies included in this review, primarily consisting of cross-sectional studies. The mean age of participants across the studies was 76 years old. The most common cause of irreversible vision loss was glaucoma, followed by age-related macular degeneration and diabetic retinopathy. Other conditions included uveitis, uveal melanoma, rhegmatogenous retinal detachment, and retinal vein occlusion. The most common scales used to measure depression were the Hospital Anxiety and Depression – Depression (HADS-D), Centre for Epidemiological Studies – Depression Scale (CES-D), and the Geriatric Depression Scale (GDS). For anxiety, the most frequently used scales were the Hospital Anxiety and Depression – Anxiety (HADS-A), State Trait Anxiety Inventory (STAI), and Generalized Anxiety Disorder 7 Item Scale (GAD-7).

**Table 1.** Study Demographics of Included Studies in Meta-Analysis and Systematic Review

Author	Year	Study design	Study location	N	Mean age	Sd	Cause of vision impairment
Banerjee	2008	Cross-sectional	India	53	69.21	8.34	Age-related macular degeneration
Brody	2002	Cross-sectional	USA	231	80.6	7.12	Age-related macular degeneration
Grant	2011	Prospective	USA	18	75.1	8.92	Age-related macular degeneration
Hayman	2007	Cross-sectional	New Zealand	391	83.7	4.8	Age-related macular degeneration
Mylona	2020	Cross-sectional	Greece	50	67	5.99	Age-related macular degeneration
Rovner	2002	Randomized control trial	USA	51	81.2	7	Age-related macular degeneration
Rovner	2014	Randomized control trial	USA	92	82.7	6.9	Age-related macular degeneration
Rovner	1997	Cross-sectional	USA	27	77.4	6.6	Age-related macular degeneration
Liu	2020	Retrospective case control	China	50 550	47.7	16.9	Age-related macular degeneration, central serous retinopathy, retinal vascular occlusion, diabetic retinopathy, glaucoma, uveitis
Kamga	2017	Randomized control trial	Canada	80	76	12	Age-related macular degeneration, diabetic retinopathy
Lamoureux	2009	Cross-sectional	Australia	103	74.7	12.2	Age-related macular degeneration, diabetic retinopathy, glaucoma
Rishi	2017	Prospective observational	India	95	38.2		Age-related macular degeneration, diabetic retinopathy, glaucoma, optic nerve disorders
Nollett	2019	Cross-sectional	UK	990	77		Age-related macular degeneration, glaucoma, diabetic retinopathy
Rees	2015	Randomized control trial	Australia	153	80.2	8.1	Age-related macular degeneration, glaucoma, diabetic retinopathy
Renieri	2013	Prospective	Germany	50	75.04	11.25	Age-related macular degeneration, glaucoma, diabetic retinopathy
Chen	2019	Retrospective cohort	Taiwan	25 939	42.9	12.3	Central serous choroidopathy
Cohen	1997	Cross-sectional	USA	49	34.3	9.2	Diabetic retinopathy
D'Amato	2016	Cross-sectional	Italy	35	59.9	8.97	Diabetic retinopathy
Hall	2017	Cross-sectional	Cameroon	261	56	12.21	Diabetic retinopathy
Kurtović	2019	Cross-sectional	Croatia	94	46.56	15.7	Diabetic retinopathy
Pan	2017	Cross-sectional	China	10	64.22	11.46	Diabetic retinopathy
Sieu	2011	Prospective cohort	USA	2359	63.9	12.9	Diabetic retinopathy
Roy	2001	Cross-sectional	USA	69	32.6	8.3	Diabetic retinopathy
Saglam	2010	Cross-sectional	Turkey	207	54.65	8.5	Diabetic retinopathy
Abe	2020	Cross-sectional	Brazil	129	70.14	15.8	Glaucoma
Agorastos	2013	Cross-sectional	Germany	69	72.7	8	Glaucoma
Ajith	2022	Prospective	India	148	62.47	9.29	Glaucoma
Cumurcu	2006	Cross-sectional	USA	113	49.65	11.11	Glaucoma
Dayal	2022	Cross-sectional	India	200	59.2	12.6	Glaucoma
Gamiochipi-Arjona	2021	Cross-sectional	Mexico	111	67.6	13.8	Glaucoma
Hollo	2009	Cross-sectional	Hungary	58	67.3	14.1	Glaucoma
Jung	2016	Cross-sectional	Korea	102	61.47	0.7	Glaucoma
Lim	2016	Cross-sectional	Singapore	100	67.1	12	Glaucoma
Mabuchi	2012	Cross-sectional	Japan	408	66.2	11.8	Glaucoma
Otori	2017	Cross-sectional	Japan	472	62.4	13.1	Glaucoma
Skalicky	2018	Prospective	Australia	101	64.7	11.1	Glaucoma
Stringham	2018	Cross-sectional	USA	74	69	9.3	Glaucoma
Tharmathurai	2021	Cross-sectional	Malaysia	360	N/A	N/A	Glaucoma
Warrian	2009	Cross-sectional	USA	148	67	10	Glaucoma
Wilson	2002	Prospective	USA	121	69.8	12.8	Glaucoma
Wu	2019	Cross-sectional	China	428	57.59	15.89	Glaucoma
Yochim	2012	Prospective	USA	41	70.0	9.2	Glaucoma
Yoshikawa	2019	Cross-sectional	Japan	770	70.9	6.9	Glaucoma
Senra	2013	Cross-sectional	Spain	38	42.7	14.5	Glaucoma, retinitis pigmentosa, retinopathy
Moschos	2018	Retrospective case control	UK	103	41	7	Keratoconus
Clancy	2022	Cross-sectional	UK	104	80.3		Macular degeneration
Crewe	2011	Cross-sectional	Australia	124	80		Macular degeneration, congenital retinal dystrophies, optic neuropathies, diabetic retinopathy, glaucoma, vein occlusions
Li	2011	Retrospective	USA	22 482	75.8	0.1	Macular degeneration, diabetic retinopathy, glaucoma
Horowitz, R, Boerner	2005	Prospective longitudinal	USA	94	76.9		Macular degeneration, glaucoma
Aa	2015	Prospective	Netherlands	265	73.7	12.3	Macular degeneration, glaucoma, cataracts, cerebral hemorrhage, diabetic retinopathy
Heesterbeek	2017	Prospective cohort	Netherlands	540	74.8	11.5	Macular degeneration, glaucoma, cataract, diabetic retinopathy, cerebral hemorrhage
Holloway	2015	Prospective longitudinal	Australia	124	77.02	9.12	Macular degeneration, glaucoma, diabetic retinopathy
Horowitz, R, Kennedy	2005	Longitudinal	USA	584	80.4	7.6	Macular degeneration, glaucoma, diabetic retinopathy
Shmuely-Dulitzki	1997	Cross-sectional	USA	70	82.5		Macular degeneration, glaucoma, diabetic retinopathy
Shmuely-Dulitzki	1995	Cross-sectional	USA	70			Macular degeneration, glaucoma, diabetic retinopathy
Do	2022	Cross-sectional retrospective	USA	293	79		Macular degeneration, glaucoma, diabetic retinopathy, retinal detachment, optic neuropathy
Canamary	2020	Cross-sectional	Brazil	81	41.5	14.5	Ocular toxoplasmosis, glaucoma, retinal detachment, cystoid macular edema, choroidal neovascular membrane
Ha	2021	Retrospective cohort	Korea	49 200	36.2		Retinal vein occlusion
Du	2019	Cross-sectional	China	116	50	13	Rhegmatogenous retinal detachment
Chabert	2004	Cross-sectional	Austria	98	63		Uveal melanoma
Eser-Öztürk	2021	Cross-sectional	Turkey	58	34.76	11.14	Uveitis
Sittivarakul	2019	Cross-sectional	Thailand	86	42.4	15.7	Uveitis, ocular inflammatory disease

### Depression in Age-Related Macular Degeneration

**Figure 2.**
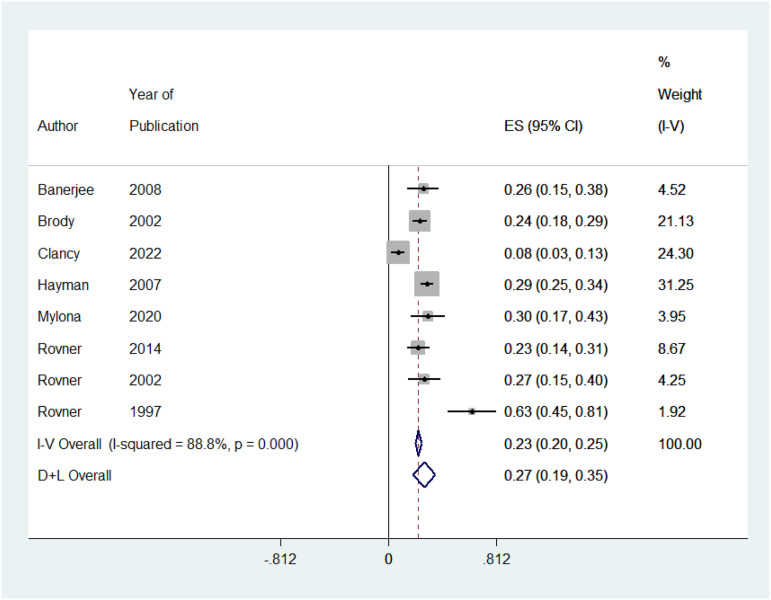
Forest Plot for Effect Size (ES) of the Prevalence of Depression in Age-Related Macular Degeneration Patients.^21-28^

The prevalence of depression in AMD patients ranged from 3% to 64% across individual studies. Figure 2. Displays the meta-analysis for the prevalence of depression, with 8 included studies. The level of heterogeneity was high between studies (I^2^ = 88.8%, *P*-value = 0.000). Therefore, a random-effects model was computed using the Dersimonian and Laird (D + L) method. In 913 patients with AMD, the prevalence of depression was 27% (95% CI.^29,30^).

### Depression in Diabetic Retinopathy

**Figure 3.**
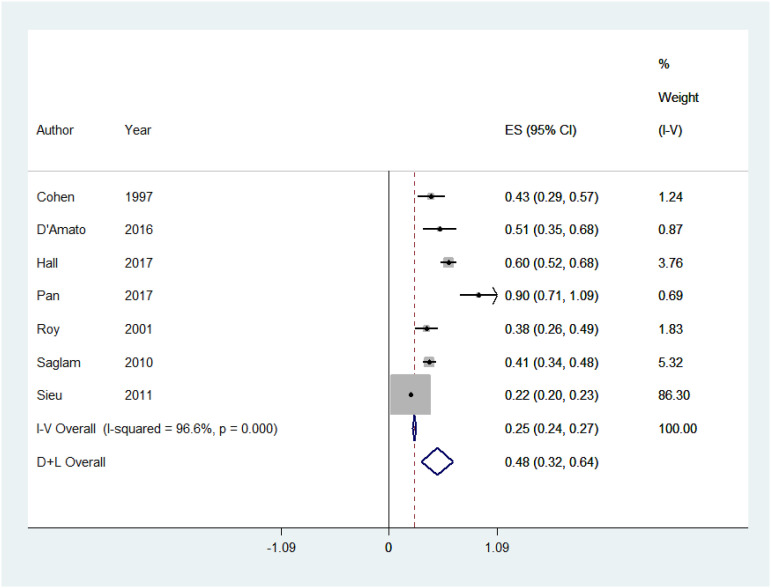
Forest Plot for Effect Size (ES) of the Prevalence of Depression in Diabetic Retinopathy Patients.^31-37^

The prevalence of depression in diabetic retinopathy patients ranged from 24% to 27% across individual studies. Figure 3. Displays the meta-analysis for the prevalence of depression, with 7 included studies. The level of heterogeneity was high between studies (I^2^ = 96.6%, *P*-value = 0.000). Therefore, a random-effects model was computed using (D + L) method. In 2990 patients with diabetic retinopathy, the prevalence of depression was 48% (95% CI).^38,39^

### Depression in Glaucoma

**Figure 4.**
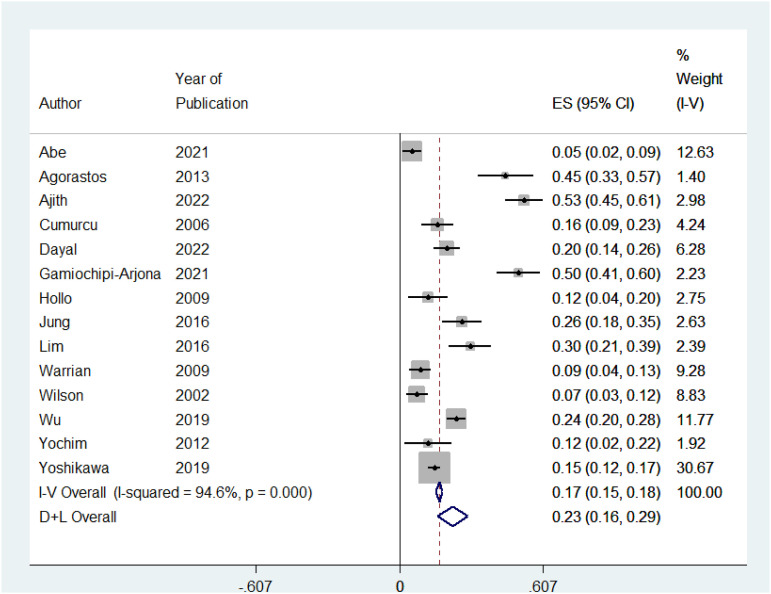
Forest Plot for Effect Size (ES) of the Prevalence of Depression in Glaucoma Patients.^30,38,40-51^

The prevalence of depression in glaucoma patients ranged from 15% to 18% across individual studies. Figure 4. Displays the meta-analysis for the prevalence of depression, with 14 included studies. The level of heterogeneity was high between studies (I^2^ = 94.6%, *P*-value = 0.000). Therefore, a random-effects model was computed using (D + L) method. In 2538 patients with glaucoma, the prevalence of depression was 23% (95% CI).^27,35^

### Depression in Irreversible Vision Loss

**Figure 5.**
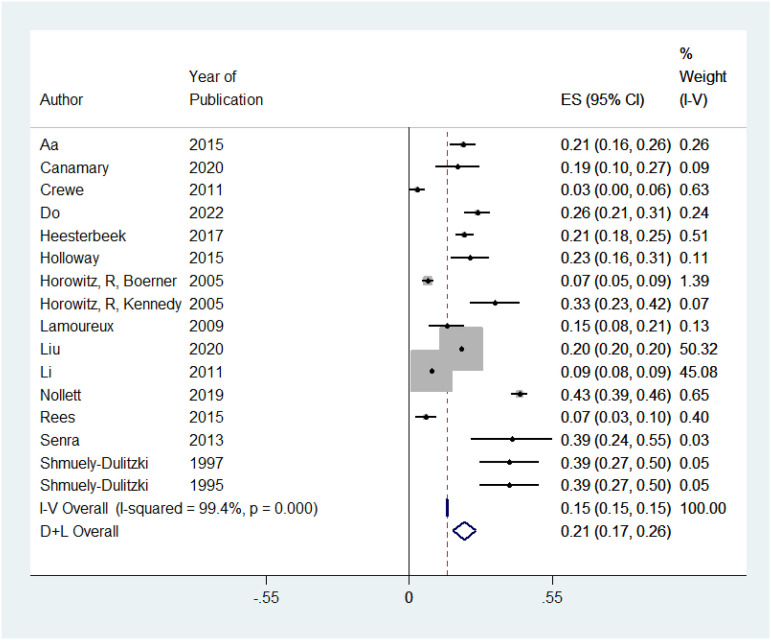
Forest Plot for Effect Size (ES) of the Prevalence of Depression in Multiple Eye Diseases Leading to Irreversible Vision Loss.^7,29,39,52-64^

The prevalence of depression in patients experiencing irreversible vision loss was 15% across individual studies. Figure 5. Displays the meta-analysis for the prevalence of depression, with 16 included studies. The level of heterogeneity was high between studies (I^2^ = 94.6%, *P*-value = 0.000). Therefore, a random-effects model was computed using (D + L) method. In 76 561 patients with irreversible vision loss, the prevalence of depression was 21% (95% CI).^33,52^

### Anxiety in Irreversible Vision Loss

**Figure 6.**
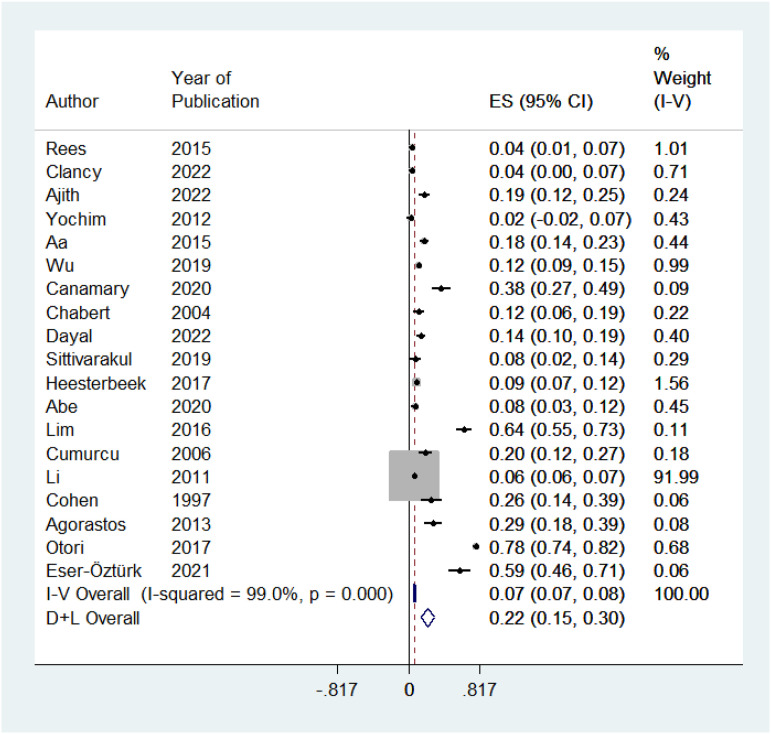
Forest Plot for Effect Size (ES) of the Prevalence of Anxiety in Multiple Eye Diseases Leading to Irreversible Vision Loss.^28,30,31,38-42,46,49,50,54,57,59,60,65-68^

The prevalence of anxiety in patients with irreversible vision loss ranged from 7 to 8% across individual studies. Figure 6. Displays the meta-analysis for the prevalence of anxiety, with 19 included studies. The level of heterogeneity was high between studies (I^2^ = 99.0%, *P*-value <0.001). Therefore, a random-effects model was computed using (D + L) method. In 25 616 patients with irreversible vision loss, the prevalence of anxiety was 22% (95% CI^26,36^). We were unable to stratify the causes of irreversible vision loss into specific disease processes due to limitations in the data available.

### Assessment of Publication Bias

**Figure 7.**
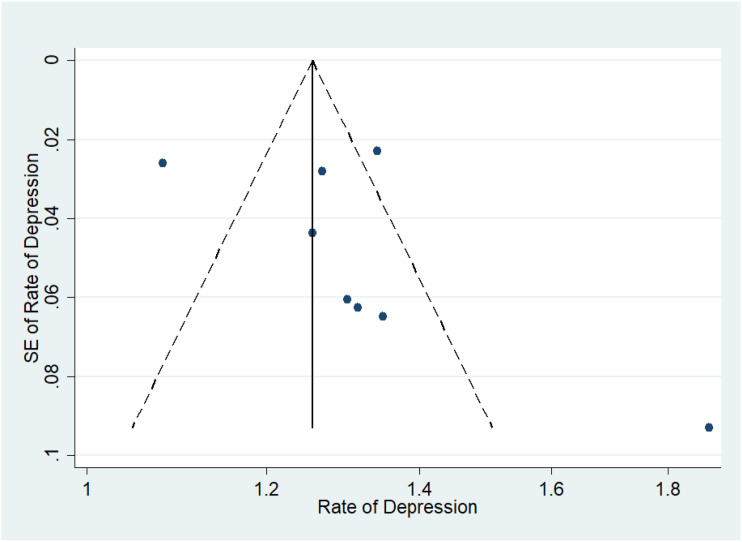
Funnel Plot to Assess Publication Bias in Studies of Depression in Age-Related Macular Degeneration Patients

**Figure 8.**
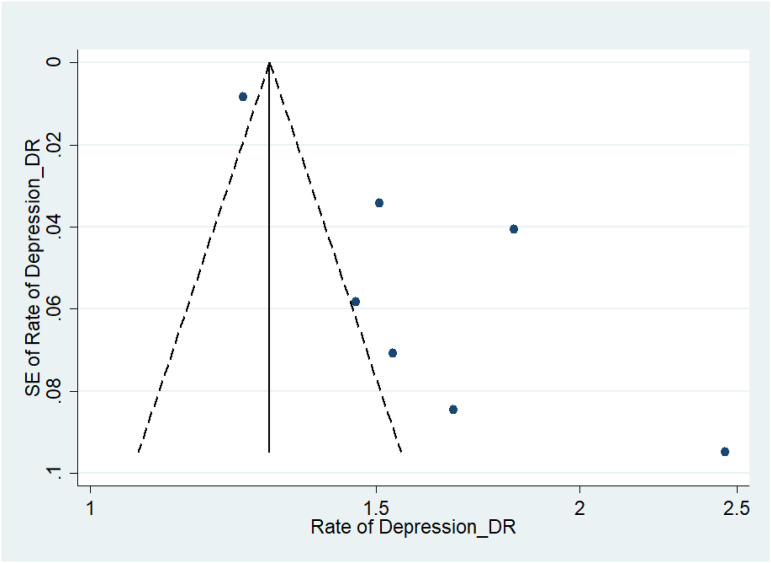
Funnel Plot to Assess Publication Bias in Studies of Depression in Diabetic Retinopathy Patients

**Figure 9.**
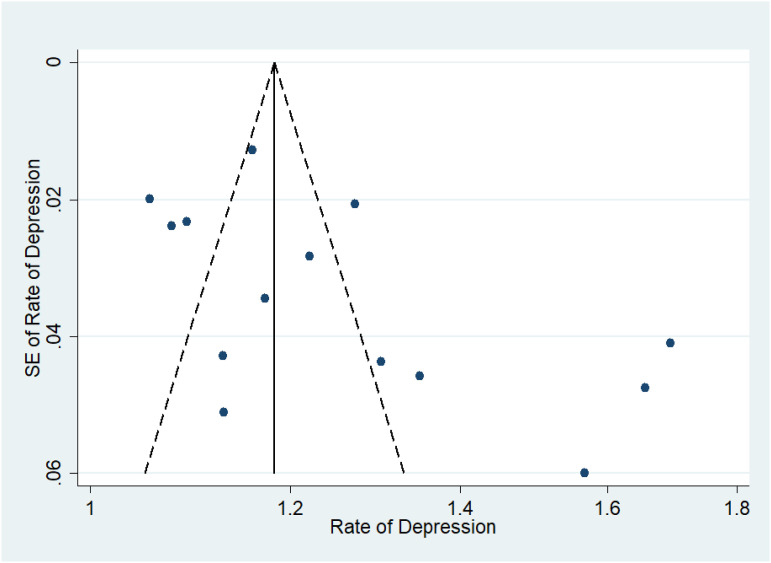
Funnel Plot to Assess Publication Bias in Studies of Depression in Glaucoma Patients

**Figure 10.**
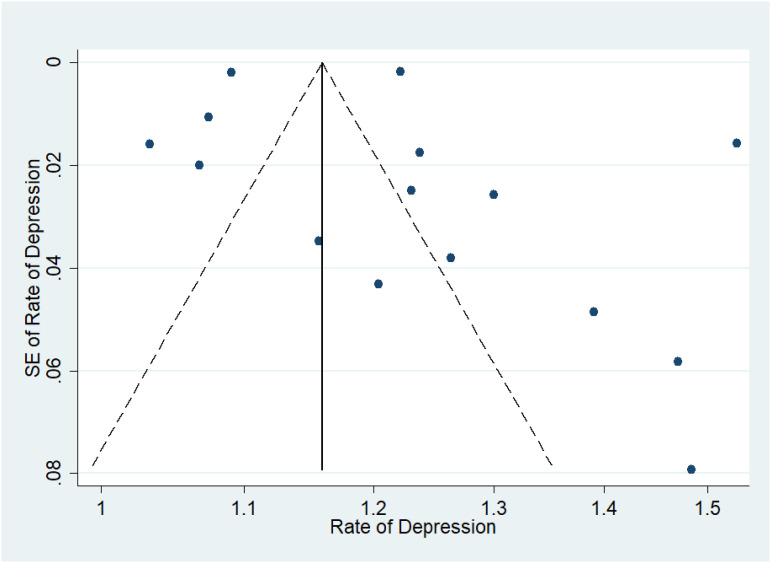
Funnel Plot to Assess Publication Bias in Studies of Depression in Multiple Eye Diseases Leading to Irreversible Vision Loss

**Figure 11.**
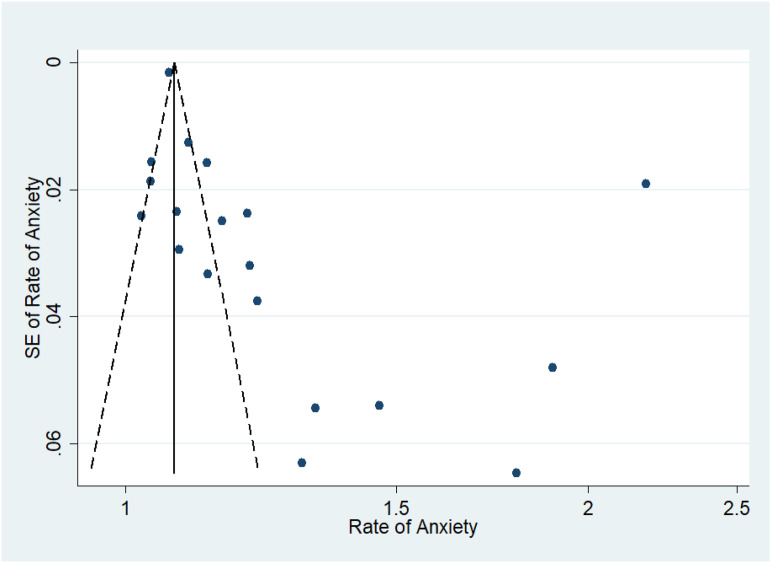
Funnel Plot to Assess Publication Bias in Studies of Anxiety in Multiple Eye Diseases Leading to Irreversible Vision Loss

Figures 7–11 represent funnel plots for various conditions in eye disease studies. Figure 7 represents funnel plots for depression in age-related macular degeneration patients, depression in diabetic retinopathy patients (Figure 8), depression in glaucoma patients (Figure 9), depression in multiple eye diseases (Figure 10), and anxiety in multiple eye diseases (Figure 11). The funnel plot has few studies at the bottom of the plot, which implies that small studies with non-significant results may not be published. Visual inspection of the funnel plot did not reveal asymmetry. Our group also performed both Egger’s and Begg’s tests to further emphasize presence of publication bias in studies examined. Egger’s test with respect to Figure 2 was insignificant (*P* = 0.26), while Begg’s test was not (*P* = 0.032). The same was true with studies included in Figure 5 (*P* = 0.35, <0.001). Both Egger’s and Begg’s test with respect to Figure 3, 4 and 6 were significant (*P* = 0.008, 0.024; *P* = 0.039, 0.007; *P* = 0.026, <0.001 respectively). Therefore, it is likely that publication bias on studies regarding this topic is present.

### Assessment of Study Quality

Based on the Modified Downs and Black Checklist,^8^ all studies obtained a score of 15 or higher, indicating “fair” or “good” quality. No studies were classified as poor quality. Our group is able to provide comprehensive information regarding the assessment of risk of bias and potential publication bias upon request.

## Discussion

This systematic review and meta-analysis included 62 studies and a total of 185 523 patients (159 352 for depression and 26 171 for anxiety) with irreversible vision loss. It aimed to investigate the relationship between irreversible vision loss and the prevalence of anxiety and depression.

In terms of evaluating the incidence of depression, we found that 21% of the 76 561 patients with irreversible vision loss experienced depression. There may be several factors that contribute to this finding. For example, the decreased quality of life related to the reduction in autonomy. Vision loss can severely impact an individual’s ability to perform daily activities independently, such as driving, and personal care.^69-71^ Difficulties with once-simple tasks often lead to dependence on others and feelings of insecurity.^72^ However, it is possible to improve QoL in individuals with irreversible vision loss. Research has shown that training programs such as trail coordination tasks and dancing improves fine motor skills, which may aid in day-to-day activities.^5^ Furthermore, use of low vision devices and group-based educational interventions resulted in fewer issues with completing activities of daily living following the training.^5^ Thus, these interventions can improve the QoL for persons with irreversible vision loss and may reduce the incidence of depression. Social isolation is another contributing factor to poor mental health as vision impairment can make it difficult to engage in social activities, leading to loneliness, which is a risk factor for depression.^73,74^ Additionally, the inability to enjoy hobbies that require adequate vision can diminish overall quality of life.^75^ The uncertainty about the progression of vision loss and its future implications can also cause significant stress, which can evolve into depression.^59^ Fortunately, some programs have shown promising results in helping alleviate this issue. Group-based education programs have shown improvements in anxiety, depression, and self-esteem.^5^ In addition, visual perception training and social skills training workshops had mixed results with some studies indicating significant improvement in social skills, but others reporting no change in social skills.^76^ Intentional and well rounded treatment plans may reduce the social isolation experienced by those with irreversible vision loss and reduce the risk of depression and anxiety.

The economic burden of vision loss may limit employment opportunities, leading to financial stress.^77^ The difficulties in adapting to a new way of living with vision impairment, while potentially requiring a career chance or the loss of work can be overwhelming.^78,79^ Adapting to visual aids and other adaptive techniques also significantly impacts mental health. This may lead to adjustment disorders and depression. Furthermore, societal stigma and personal perceptions of being “disabled” can affect self-esteem, leading to social withdrawal and depression.^80^ When considering the link between depression and specific eye diseases such as diabetic retinopathy, age-related macular degeneration (AMD), and glaucoma, unique factors and challenges come into play. Among those with diabetic retinopathy, the prevalence of depression was 48%, notably the highest rate of patients with low vision experiencing any mental health impairment. This high prevalence is likely due to the comorbidities associated with diabetes, including neuropathy, nephropathy, and cardiovascular disease.^81-83^ End-stage complications manifest as neuropathic pain, end-stage renal disease, and even limb amputation.^84-86^ Managing diabetic retinopathy requires frequent medical appointments, and invasive treatments, including regular anti-VEGF injections.^87,88^ This may result in treatment fatigue and emotional strain. Furthermore, patients must adhere to strict lifestyle changes to manage their diabetes, which can be challenging.^89,90^ This higher burden of disease may explain why those with diabetic retinopathy experience worse depression than other causes of irreversible vision loss.

In comparison, the prevalence of depression was 27% among those with AMD which primarily affects older adults, who may already be dealing with other health issues, increasing their vulnerability to depression. These issues include physical health decline, cognitive decline, and social isolation.^91,92^ The loss of central vision critically impacts activities such as reading, recognizing faces, and driving, leading to significant lifestyle limitations.^93,94^ The progressive nature of AMD can cause anxiety and a constant fear of worsening vision contributes to depression.

Comparatively, for those with glaucoma, the prevalence of depression was 23%, the lowest among the conditions studied. This is likely because central vision is usually spared until the late stages of the disease and thus functional independence may be spared till later for things such as reading and facial recognition. However, the chronic nature of glaucoma requires lifelong management, which can be mentally exhausting and lead to chronic stress and depression.^91,92^ Additionally, the medications used to manage glaucoma can have side effects that affect mood and overall well-being, contributing to depressive symptoms.^91,93^ Lastly, peripheral vision loss associated with glaucoma may lead to difficulties with mobility and an increased risk of accidents, increasing fear.^94,95^

Our research shows that physiological conditions that result in irreversible vision loss have detrimental impacts on functioning, anxiety, socializing, and finances. These can all contribute to higher rates of depression in these populations. However, there are methods to improve these factors. For example, tailored interventions such as low vision devices, psychological support, and patient education can help mitigate depression and improve the quality of life for patients with vision loss.^96^ Thus, understanding these unique challenges is essential for effective management. In terms of anxiety, the study demonstrated a prevalence of 22% for anxiety among 25 616 patients with irreversible vision loss. Anxiety in these patients is driven by various factors such as the fear of further vision deterioration, and the challenges of adapting to visual impairment.^97^ These challenges can cause emotional stress, which may significantly compromise their QoL. Furthermore, uncertainty about the progression of vision loss can cause significant anxiety, as patients worry about the potential for further blindness and its impact on their lives.^98^ As mentioned previously, vision loss severely affects the ability to perform daily activities independently, such as driving, and hygiene, leading anxiety about being a burden on others.^99^ Learning to use visual aids and adaptive techniques can be stressful, causing anxiety about their effectiveness and reliability. Lastly, managing vision loss often requires frequent medical appointments and complex treatment regimens, which can be anxiety-inducing, especially if patients fear the outcomes of their treatments.^100-103^

In terms of improving anxiety in those with vision loss, there are some promising results. Studies have shown that using low vision devices, along with education such as self-management techniques and behavioral, can help with the treatment of depression and anxiety.^103^ Furthermore, having individuals with irreversible vision loss attend cognitive behavioral therapy sessions has been shown to reduce anxiety.^103^ Lastly, general therapies used for anxiety in the general population such as lifestyle modifications like exercise or meditation may be helpful in those with vision impairment as well.^96^ Thus, given the high prevalence of anxiety and depression among patients with irreversible vision loss, healthcare providers must be vigilant in screening for these conditions. Integrating mental health support into the routine care of patients with vision loss is crucial. This support can include psychotherapy, and, where appropriate, pharmacological interventions. Of course, lifestyle modifications such as exercise, social support, and stress-reduction techniques should also be encouraged to improve physical mobility and psychological well-being.

## Study Limitations

The primary limitation was our inability to stratify the data regarding prevalence rates of anxiety into specific diseases, as we were able to do with depression. Many of the studies investigating anxiety looked at patients with multiple diseases,^39,49,54,60,68^ and were therefore unable to be analyzed with respect to individual diseases and their correlation with rates of anxiety. As different comorbidities can have varying impacts on the prevalence rates of anxiety,^104^ it is likely that further subgroup analysis would have yielded more valuable information, therefore highlighting the importance of future research focusing on patients with specific disease which cause irreversible vison loss.

A secondary limitation of our research was the presence of significant heterogeneity across most pooled estimates, which raises concerns about the pooling validity. The most likely reason for heterogeneity is the lack of control for moderators such as sex, age, other comorbidities, disease stage, and reporting scales. Previous studies have indicated that these are all factors which may influence the prevalence rates of mental illness.^105,106^ Therefore, included studies lacking control for these factors is likely the reason for observed heterogeneity. As previously mentioned, the high level of heterogeneity among the studies indicates the need for more standardized research methods in this field.

Poor transparency of data with regard to prevalence rates of anxiety in patients with irreversible vision loss and high levels of heterogeneity between studies also created a challenge in assessing which studies should be included in the final analysis – hence the Cohen’s Kappa value of 21%. Improving the quality of research relating mental health occurrence to mental health conditions causing vison loss via standardization of data will also improve the ability of researchers conducting systematic reviews to identify which studies are most relevant on for topic.

## Conclusion

Significant prevalence of depression and anxiety among patients with irreversible vision loss highlights the critical need for comprehensive healthcare approaches that address both physical and mental health. By integrating mental health screening and support into the care of patients with vision loss, healthcare providers can improve overall outcomes and QoL for this vulnerable population. Screenings and interventions integrated across a variety of medical specialties can support an interdisciplinary care team approach to managing the overall well-being of patients. Future research should investigate what factors are protective against the development of anxiety and depression. In addition, researchers should focus on the long-term effects of targeted vision loss treatments on mental health outcomes and the development of targeted interventions. Studies investigating whether interventions for vision loss, such as anti-VEGF therapy for AMD, can concurrently improve symptoms of depression and anxiety would be particularly valuable. In addition, understanding the mechanisms underlying the relationship between vision loss and mood disorders may be useful in developing more targeted interventions.
